# Effectiveness of acupuncture for nocturia: A protocol for systematic review and meta-analysis

**DOI:** 10.1097/MD.0000000000025739

**Published:** 2021-05-21

**Authors:** Yingjie Nie, Yushan Fan, Lilin Huang, Xiaojun Zhao, Ruikang Pang, Yijia Yang

**Affiliations:** a Guangxi University of Chinese Medicine; b College of Acupuncture and Massage, Guangxi University of Chinese Medicine, Nanning, China.

**Keywords:** acupuncture, complementary therapy, nocturia, randomized controlled trial, systematic review

## Abstract

**Background:**

Nocturia is a common and highly troubled lower urinary tract symptom, which has a wide range of effects. About 33% of patients with lower urinary tract symptoms have been affected by nocturia. Nocturia is mainly manifested as the increase of urination frequency and urine volume at night. It has been proved that acupuncture can reduce the symptoms of nocturia and regulate bladder function in Western countries. Acupuncture may be a promising choice for the treatment of nocturia.

**Methods:**

RCTs of acupuncture for nocturia will be searched in the relevant database, including PubMed, Embase, Cochrane Library, China National Knowledge Infrastructure Wanfang Database, Chinese Biomedical Literature Database, and Chinese Scientific Journal Database. The studies of electronic searches will be exported to EndNote V.9.1 software. We will run meta-analyses using the Review Manager (RevMan) V.5.3 software. Any disagreement will be solved in consultation with a third reviewer.

**Results:**

Our study aims to explore the efficacy of acupuncture for nocturia and to provide up-to-date evidence for clinical of nocturia.

**Conclusion:**

The conclusion of this study will provide evidence for the efficacy of acupuncture treatment of nocturia.

**INPLASY registration number:**

INPLASY202130100.

## 1 Introduction

Nocturia is a common and highly troubling condition of the lower urinary tract characterized by increased frequency and volume of urination at night.^[1]^ The International Continence Society^[2]^ defines it as “the complaint that the individual has to wake at night 1 or more times to void.” Nocturia is widespread in adults, and approximately 33% of patients with lower urinary tract symptoms show that they are affected by nocturia. A retrospective study of the prevalence of nocturia from 1990 to 2009 found that among young adults aged 20 to 40 years, the incidence ranged from 11% to 35.2% in males and 20.4% to 43.9% in females, while the prevalence ranged from 68.9% to 93% in older adults aged 70 years and older.^[3]^

According to the pathophysiological mechanism of nocturia, its etiology is mainly caused by 4 aspects: nocturnal polyuria, 24-hour polyuria, decreased bladder volume and sleep disturbance.^[4]^ Its occurrence and development are closely related to bladder hyperactivity and lower urinary tract symptoms,^[5]^ The essence is the result of an imbalance between functional bladder storage capacity and urine production.^[6]^ Nocturia is not only characterized by increased nocturnal urination, but also may be associated with the risk of other diseases. Frequent nocturnal awakenings due to nocturia can lead to worse sleep, increased daytime sleepiness, and reduced energy and activity in older adults.^[7]^ Older adults are also at increased risk of falling due to repeated night time bathroom visits, Datas show that,^[8]^ 25% of falls in the elderly are directly related to nocturia, and patients who use the night time bathroom at least two or more times per night more than double the risk of fractures and fall-related trauma. Nocturia is also associated with an increased risk of urinary tract disorders such as urethral obstruction, bladder diverticulum, hydronephrosis, and vesicoureteral reflux, as well as an increased prevalence of chronic diseases such as hyperglycemia, diabetes, coronary heart disease, and nervous system.^[9–11]^ It is accompanied by a variety of complications seriously affect the physiological and psychological state of patients, reduce the quality of life (N-QoL) of individuals.^[12]^ Currently, nocturia is mainly treated with drugs, including desmopressin, α1 receptor antagonists, 5α reductase inhibitors, and antimuscarinic drugs.^[13,14]^ These drugs mainly improve nocturia symptoms by enhancing the kidney's reabsorption of water during the night,^[15]^ regulate the activity of the brainstem urination center^[16]^ and improve urodynamic^[17]^ However, drugs often have side effects during treatment. For example, alpha 1 blockers can lead to cardiovascular disease, aggravate heart failure, induce vasodilation-induced lowering of blood pressure, lead to postostatic hypotension, and cause dizziness and falls.^[18]^ Desmopressin tends to cause hyponatremia,^[19]^ and 5-ARI can worsen erectile dysfunction and lower testosterone levels in men.^[20]^ Although some therapeutic measures such as lifestyle regulation, Magnetic stimulation, and botulinum toxin A (BoNT/A) have been used to promote the improvement of nocturia symptoms,^[21–23]^ the efficacy of these treatments is relatively limited and there is A lack of efficacy data,^[24]^ so the treatment of nocturia still needs to be further improved.

Acupuncture, as a traditional Chinese medicine therapy, is a kind of nerve stimulation technology. In clinical practice, it can produce percutaneous tibial posterior stimulation^[25]^ and sacral nerve regulation through acupuncture and moxibustion treatment,^[26]^ forming electrical nerve effect,^[27]^ so as to play a therapeutic role. At present, acupuncture has been widely used in the treatment of nocturia. An increasing number of clinical studies have shown that acupuncture can reduce the number of nocturia and nocturnal urine volume, and help improve patients’ bladder function and lower urinary tract symptoms.^[28–31]^ Although there are some studies on acupuncture and moxibustion in the treatment of nocturia, the meta-analysis of acupuncture in the treatment of nocturia is still relatively blank. Therefore, we will use all relevant randomized controlled trials (RCTs) data for meta-analysis to comprehensively evaluate the clinical efficacy of acupuncture and moxibustion in the treatment of nocturia.

## 2 Objectives

The aims are:

1. to explore the efficacy of acupuncture for nocturia and
2. to provide up-to-date evidence for clinical of nocturia.

## 3 Methods and analysis

### 3.1 Study registration

The protocol for this review was developed in accordance with the PRISMA-P guidelines and the Cochrane Handbook.^[32,33]^ This protocol has been registered on INPLASY (registration number: INPLASY202130100: https://inplasy.com/inplasy-2021-3-0100/).

### 3.2 Inclusion criteria

#### 3.2.1 Type of studies

All RCTs reported will be included without regional and language restrictions. Animal studies, cohort studies, case-controlled studies, case reports, and expert experience will be excluded.

#### 3.2.2 Type of participants

Participants in the study were diagnosed with nocturia. On the basis of International Continence Society -2002 report on standardization of terminology in nocturia^[2]^ “the complaint that the individual has to wake at night 1 or more times to void” regardless the age, gender, race, country, and nocturia type.

#### 3.2.3 Type of interventions

The purpose of this study is to observe the clinical study of acupuncture in the treatment of nocturia. Acupuncture treatment were used in the experiment, including body acupuncture, warm acupuncture, electro-acupuncture, auricular acupuncture, fire needling, elongated needle, moxibustion, or herbs-partitioned moxibustion.

#### 3.2.4 Type of comparators

The control group that will include nonacupuncture techniques, such as behavioral therapy, sham acupuncture, placebo, or pharmacotherapy. The acupoint numbers, retaining time, and frequency will not be restricted in this protocol.

#### 3.2.5 Type of outcome measures

##### 3.2.5.1 Primary outcomes

The primary outcome measures will be total and frequency of nocturnal urination.

##### 3.2.5.2 Secondary outcomes

Secondary outcomes will include the ratio of nocturnal urine volume to daytime urine volume. Urine/blood osmotic pressure ratio, urine specific gravity value. change in N-QoL, Pittsburgh Sleep Quality Index Scale, as well as a standard battery of blood and urine analyses, vital signs, and physical examinations.

### 3.3 Exclusion criteria

- Non-RCTs;
- None of the valid outcome indicators;
- Duplicated data;
- Invalid outcome indexes.

### 3.4 Search methods for identification of studies

#### 3.4.1 Electronic searches

RCTs of acupuncture for nocturia will be searched in the relevant database, including PubMed, Embase, Cochrane Library, China National Knowledge Infrastructure Wanfang Database, Chinese Biomedical Literature Database, and Chinese Scientific Journal Database. The key words include “acupuncture,” “nocturia,” “complementary therapy,” “randomized controlled trial,” “randomized controlled trial” and “systematic review.” An equivalent translation of the same search terms will be used to search in the Chinese databases. The search strategy of PubMed is shown in Table 1.

**Table 1:** Search strategy used in PubMed database.

Order	Search items
#1	(Nocturia[MeSH Terms]) AND (Nocturia)
#2	(((((((((((((((((Acupuncture[MeSH Terms]) OR (Pharmacopuncture)) OR (Acupuncture therapy)) OR (Electroacupuncture)) OR (Manual acupuncture)) OR (Dry Needle)) OR ((Moxibustion[MeSH Terms]) OR (moxibustion))) OR (Acupuncture, Ear[MeSH Terms])) OR (Acupuncture, ear)) OR (Ear acupuncture)) OR (Auricular acupuncture)) OR (Ear acupuncture)) OR (Acupuncture, auricular)) OR (Acupuncture, auricular)) OR (Auricular acupuncture)) OR (Warm acupuncture)) OR (Elongated needle)
#3	Randomized controlled trial[Publication Type] OR Randomized[Title/Abstract] OR placebo[Title/Abstract]
#4	#1 AND #2 AND #3

#### 3.4.2 Searching other resources

A review or meta-analysis of relevant RCT systems will be conducted via electronic search. We will also manually search the references of relevant articles that is not included in the electronic database to further identify eligible studies.

### 3.5 Selection of studies

**Figure 1:**
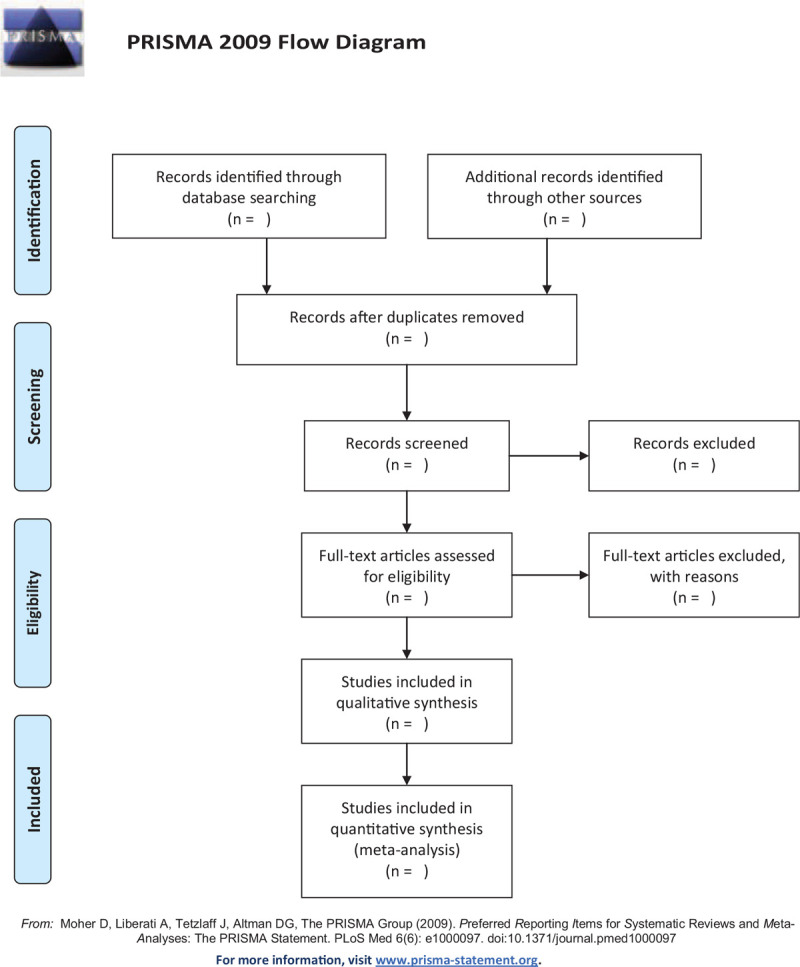
Flowchart of literature selection.

The studies of electronic searches will be exported to EndNote V.9.1 software. Two authors will independently undertake the process of selecting the search results according to the inclusion and exclusion criteria. They will review and screen the titles and abstracts retrieved by literature search to exclude irrelevant trials. The causes of both selections will be documented and full texts will be obtained and checked for further evaluation if necessary. When there is uncertainty about eligibility of the study, reviewers will arrive at a decision by via discussion and consensus with a third reviewer. The selection process will be showed in a PRISMA flow diagram (Fig. 1).

### 3.6 Data extraction and management

Data will be extracted independently from the selected articles by 2 reviewers using a Microsoft Excel spreadsheet. They will review and screen the titles and abstracts retrieved by literature search to exclude irrelevant trials and cross-check. If there is any disagreement, discuss and resolve it and consult the third party for assistance in judgment. Data extraction including:

1. author, year, random method, sample size, age;
2. specific details of the intervention, treatment, amount of treatment, duration of intervention, acupoint selection, and course of treatment;
3. outcome indicators and outcome measurement data of concern.

Disagreements between reviewers in the process of data extraction were resolved by discussing with a third reviewer. Incomplete data will be provided by contacting corresponding authors.

### 3.7 Assessment of the methodological quality

The risk of deviation was assessed by 2 reviewers against criteria provided in the Cochrane Intervention Systematic Evaluation Manual.^[33]^ It includes the following 7 domains: random sequence generation, allocation concealment, blinding of participants and personnel, blinding of outcome assessment, incomplete outcome data, selective reporting. Any disagreement should be solved in consultation with a third reviewer.

### 3.8 Measures of treatment effect

Weighted mean difference or standardized mean difference will be adopted as statistical indicators in the analysis of continuous outcomes and the relative risk will be used to assess the treatment effect for dichotomous outcomes. 95% of the confidence intervals will be determined in pooled estimates.

### 3.9 Dealing with missing data

We will attempt to contact authors to obtain missing data. If we cannot contact the original authors, the studies will be excluded from the data synthesis.

### 3.10 Assessment of heterogeneity

Statistical heterogeneity should be evaluated by Chi-Squared tests and *I*^*2*^ statistic. The results of the *I*^*2*^ statistic, which determine the using of fixed-effects model or random-effects model, cover unimportant heterogeneity (0%–40%), moderate heterogeneity (30%–60%), substantial heterogeneity (50%–90%), and considerable heterogeneity (75%–100%). A random-effect model or subgroup analysis should be used when there exists significant heterogeneity.

### 3.11 Data synthesis

We will run meta-analyses using the Review Manager (RevMan) V.5.3 software. If the result of heterogeneity in *I*^*2*^ < 40%, the fixed-effects model will be used for data synthesis and analysis; If *I*^*2*^ ≥ 40% and < 75%, the random-effects model will be implied; If *I*^*2*^ ≥ 75%, it means there is considerable heterogeneity between studies. Alternatively, we will eliminate low-quality research and use sensitivity analysis to investigate which studies will be the most likely have a significant effect on heterogeneity. If synthesize quantitatively is not possible, we’re going to do a qualitative description.

### 3.12 Subgroup analysis

If there is significant heterogeneity in the results, we will conduct a subgroup analysis to investigate differences in age, gender, length of disease duration, outcome style, etc.

#### 3.12.1 Sensitivity analysis

We will perform sensitivity analyses to verify robustness of results. It includes the impact of methodological quality, study design, and sample size.

### 3.13 Grading the quality of evidence

Two reviewers will independently use the Grading of Recommendations Assessment, Development and Evaluation, scored each criterion as having high risk, low risk or unclear risk.

### 3.14 Ethics and dissemination

The study will be published in peer-reviewed journals or relevant conferences. Ethical approval is not required. The results of this study will provide potential guidance for promoting the treatment strategy of nocturia patients.

## 4 Discussion

The frequent nocturnal urination caused by nocturia can negatively affect patients’ sleep quality, mental state, and N-QoL.^[34]^ Many beneficial explorations on nocturia have been made around the world, which have made many important contributions to the understanding of etiology and pathology of nocturia as well as the prevention and treatment of the disease, and provided a variety of options for improving the N-QoL of patients with nocturia. However, due to the characteristics of recurrent nocturia and lingering difficulty in healing, the current treatment is difficult to achieve long-term good control of the symptoms of patients, and the safety of its prognosis and efficacy is still not ideal. Therefore, there is an urgent need for a stable, safe, and convenient treatment. Therefore, there is an urgent need for a stable, safe and convenient treatment. Acupuncture, as a traditional Chinese medicine therapy, is also increasingly widely used in the treatment of nocturia. Several RCT studies recently have also provided relevant evidence to estimate the effectiveness of acupuncture for treating patients with nocturia. But now is still lack of enough according to support to get a clear conclusion, therefore this study for the first time to a systematic review and meta-analysis of available literature, objectively evaluate the clinical efficacy of acupuncture in the treatment of nocturia, acupuncture treatment for the future nocturia provide objective statistics, and for clinicians treating nocturia increased reliable reference, benefit the patients.

## Author contributions

**Conceptualization:** Yingjie Nie, Yushan Fan.

**Data curation:** Yingjie Nie, Lilin Huang, Xiaojun Zhao.

**Formal analysis:** Lilin Huang, Xiaojun Zhao, Ruikang Pang.

**Methodology:** Ruikang Pang, Yijia Yang.

**Software:** Xiaojun Zhao, Ruikang Pang, Yijia Yang.

**Supervision:** Yushan Fan, Lilin Huang.

**Writing – original draft:** Yingjie Nie, Ruikang Pang, Yijia Yang.

**Writing – review & editing:** Yingjie Nie, Yushan Fan, Xiaojun Zhao.

## References

[1] Bliwise DL Wagg A Sand PK. Nocturia: a highly prevalent disorder with multifaceted consequences. Urology 2019;133:03–13. 10.1016/j.urology.2019.07.005 31310770

[2] van Kerrebroeck P Abrams P Chaikin D. The standardization of terminology in nocturia: report from the Standardization Sub-committee of the International Continence Society. Neurourol Urodyn 2002;21:179–83. 11857672 10.1002/nau.10053

[3] Bosch JL Weiss JP. The prevalence and causes of nocturia. J Urol 2013;189: 1 Suppl: S86–92. 23234639 10.1016/j.juro.2012.11.033

[4] Yazici CM Kurt O. Combination therapies for the management of nocturia and its comorbidities. Res Reports Urol 2015;7:57–63. 10.2147/RRU.S51140 PMC4408945 25945323

[5] Bliwise DL Foley DJ Vitiello MV. Nocturia and disturbed sleep in the elderly. Sleep Med 2009;10:540–8. 18703381 10.1016/j.sleep.2008.04.002 PMC2735085

[6] Olesen T Paul J Gramme P. Assessment of the most impactful combination of factors associated with nocturia and to define nocturnal polyuria by multivariate modelling. J Clin Med 2020;9: 10.3390/jcm9072262 PMC7408683 32708764

[7] Asplund R. Nocturia, nocturnal polyuria, and sleep quality in the elderly. J Psychosom Res 2004;56:517–25. 15172208 10.1016/j.jpsychores.2004.04.003

[8] Leslie SW Sajjad H Singh S. Nocturia StatPearls. Treasure Island (FL): StatPearls Publishing Copyright (2021, StatPearls Publishing LLC); 2021.

[9] Madhu C Coyne K Hashim H. Nocturia: risk factors and associated comorbidities; findings from the EpiLUTS study. Int J Clin Pract 2015;69:1508–16. 26351086 10.1111/ijcp.12727

[10] Lightner DJ Krambeck AE Jacobson DJ. Nocturia is associated with an increased risk of coronary heart disease and death. BJU Int 2012;110:848–53. 22233166 10.1111/j.1464-410X.2011.10806.x PMC3508707

[11] Bliwise DL Howard LE Moreira DM. Nocturia and associated mortality: observational data from the REDUCE trial. Prostate Cancer Prostatic Dis 2019;22:77–83. 30214036 10.1038/s41391-018-0090-5

[12] Rose GE Denys MA Kumps C. Nocturnal voiding frequency does not describe nocturia-related bother. Neurourol Urodyn 2019;38:1648–56. 31165518 10.1002/nau.24029

[13] Sakalis VI Karavitakis M Bedretdinova D. Medical treatment of nocturia in men with lower urinary tract symptoms: systematic review by the European Association of urology guidelines panel for male lower urinary tract symptoms. Eur Urol 2017;72:757–69. 28666669 10.1016/j.eururo.2017.06.010

[14] Andersson KE Van Kerrebroeck P. Pharmacotherapy for nocturia. Curr Urol Rep 2018;19:08. 10.1007/s11934-018-0750-y PMC5807446 29427214

[15] Han J Jung JH Bakker CJ. Desmopressin for treating nocturia in men. Cochrane Database Syst Rev 2017;10:Cd012059. 29055129 10.1002/14651858.CD012059.pub2 PMC6485329

[16] Hajdinjak T Leskovar J. Comparison of nocturia response to desmopressin treatment between patients with normal and high nocturnal bladder capacity index. ScientificWorldJournal 2013;2013:878564. 24223034 10.1155/2013/878564 PMC3816078

[17] Zhang HL Huang ZG Qiu Y. Tamsulosin for treatment of lower urinary tract symptoms in women: a systematic review and meta-analysis. Int J Impot Res 2017;29:148–56. 28424499 10.1038/ijir.2017.12

[18] Akinaga J García-Sáinz JA A SP. Updates in the function and regulation of (1) -adrenoceptors. Br J Pharmacol 2019;176:2343–57. 30740663 10.1111/bph.14617 PMC6592863

[19] Fralick M Schneeweiss S Wallis CJD. Desmopressin and the risk of hyponatremia: a population-based cohort study. PLoS Med 2019;16:e1002930. 31634354 10.1371/journal.pmed.1002930 PMC6802819

[20] Schröder F Bangma C Angulo JC. Dutasteride treatment over 2 years delays prostate-specific antigen progression in patients with biochemical failure after radical therapy for prostate cancer: results from the randomised, placebo-controlled Avodart After Radical Therapy for Prostate Cancer Study (ARTS). Eur Urol 2013;63:779–87. 23176897 10.1016/j.eururo.2012.11.006

[21] van Kerrebroeck P Hashim H Holm-Larsen T. Thinking beyond the bladder: antidiuretic treatment of nocturia. Int J Clin Pract 2010;64:807–16. 20337753 10.1111/j.1742-1241.2010.02336.x

[22] Apostolidis A Rahnama’i MS Fry C. Do we understand how botulinum toxin works and have we optimized the way it is administered to the bladder? ICI-RS 2014. Neurourol Urodyn 2016;35:293–8. 26872570 10.1002/nau.22797

[23] Olivera CK Meriwether K El-Nashar S. Nonantimuscarinic treatment for overactive bladder: a systematic review. Am J Obstet Gynecol 2016;215:34–57. 26851599 10.1016/j.ajog.2016.01.156

[24] Nambiar AK Bosch R Cruz F. EAU guidelines on assessment and nonsurgical management of urinary incontinence. Eur Urol 2018;73:596–609. 29398262 10.1016/j.eururo.2017.12.031

[25] Peters KM Carrico DJ Perez-Marrero RA. Randomized trial of percutaneous tibial nerve stimulation versus Sham efficacy in the treatment of overactive bladder syndrome: results from the SUmiT trial. J Urol 2010;183:1438–43. 20171677 10.1016/j.juro.2009.12.036

[26] Raju R Linder BJ. Evaluation and treatment of overactive bladder in women. Mayo Clin Proc 2020;95:370–7. 32029089 10.1016/j.mayocp.2019.11.024

[27] Abello A Das AK. Electrical neuromodulation in the management of lower urinary tract dysfunction: evidence, experience and future prospects. Ther Adv Urol 2018;10:165–73. 29623108 10.1177/1756287218756082 PMC5881994

[28] Mak TC Chen HY Cho WC. Acupuncture for overactive bladder in adults: a systematic review and meta-analysis. Acupunct Med 2019;37:321–31. 31433197 10.1136/acupmed-2017-011528

[29] Yuan H Wei N Li Y. Effect of depth of electroacupuncture on the IPSS of patients with benign prostatic hyperplasia. Evid Based Complement Alternat Med 2019;2019:1439141. 31915442 10.1155/2019/1439141 PMC6930788

[30] Zhang T Xun YQ Li B. Effect of fire needle therapy on mild-moderate benign prostatic hyperplasia: protocol for a randomized controlled pilot trial. Medicine (Baltimore) 2020;99:e20376. 32481334 10.1097/MD.0000000000020376 PMC7249880

[31] Lee HY Yun YJ Choi JY. Effectiveness and safety of moxibustion for alleviating symptoms of overactive bladder: a prospective, randomized controlled, crossover-design, pilot study. Medicine (Baltimore) 2018;97:e12016. 30142847 10.1097/MD.0000000000012016 PMC6113034

[32] Shamseer L Moher D Clarke M. Preferred reporting items for systematic review and meta-analysis protocols (PRISMA-P) 2015: elaboration and explanation. BMJ 2015;350:g7647. 25555855 10.1136/bmj.g7647

[33] Higgins JP Altman DG Gøtzsche PC. The Cochrane Collaboration's tool for assessing risk of bias in randomised trials. BMJ 2011;343:d5928. 22008217 10.1136/bmj.d5928 PMC3196245

[34] Choi EPH Wan EYF Kwok JYY. The mediating role of sleep quality in the association between nocturia and health-related quality of life. Health Qual Life Outcomes 2019;17:181. 31829192 10.1186/s12955-019-1251-5 PMC6907224

